# Evidence-Based Cesarean Delivery for the Nonobstetrician

**DOI:** 10.1055/s-0035-1570316

**Published:** 2015-12-18

**Authors:** Joshua D. Dahlke, Hector Mendez-Figueroa, Jeffrey D. Sperling, Lindsay Maggio, Brendan D. Connealy, Suneet P. Chauhan

**Affiliations:** 1 Division of Maternal-Fetal Medicine, Nebraska Methodist Women's Hospital and Perinatal Center, Omaha, Nebraska, United States; 2 Division of Maternal-Fetal Medicine, Department of Obstetrics, Gynecology, and Reproductive Sciences, UT Health-University of Texas Medical School at Houston, Texas, United States; 3 Department of Obstetrics and Gynecology, Warren Alpert Medical School of Brown University, Women and Infants Hospital of Rhode Island, Providence, Rhode Island, United States; 4 Division of Maternal-Fetal Medicine, Department of Obstetrics and Gynecology, Morsani College of Medicine, University of South Florida, Tampa, Florida, United States

**Keywords:** cesarean delivery, surgical technique, evidence-based

## Abstract

Cesarean delivery (CD) is one of the most common major surgeries performed in the United States and worldwide. Surgical techniques evaluated in well-designed randomized controlled trials (RCTs) that demonstrate maternal benefit should be incorporated into practice. The objective of this review is to provide a summary of surgical techniques of the procedure and review the evidence basis for them for the nonobstetrician. The following techniques with the strongest evidence should be commonly performed, when feasible: (1) prophylactic antibiotics with a single dose of ampicillin or first-generation cephalosporin prior to skin incision; (2) postpartum hemorrhage prevention with oxytocin infusion of 10 to 40 IU in 1 L crystalloid over 4 to 8 hours; (3) low transverse skin incision; (4) blunt or sharp subcutaneous and fascial expansion; (5) blunt, cephalad–caudad uterine incision expansion; (6) spontaneous placental removal; (7) blunt-tip needle usage during closure; (8) subcutaneous suture closure (running or interrupted) if thickness is ≥2 cm; and (9) skin closure with suture. Although the number of RCTs designed to optimize maternal and neonatal outcomes of this common procedure is encouraging, further work is needed to minimize surgical morbidity. Optimal methods for postpartum hemorrhage prevention, adhesion prevention, and venous thromboembolism prophylaxis remain ongoing areas of active research, with outcomes that could markedly improve maternal morbidity and mortality. If evidence of a surgical technique appears preferred over another, clinicians should be comfortable adopting the evidence-based technique when performing and teaching CD.

Approximately 1.3 million women undergo cesarean delivery (CD) annually in the United States, making it the most common major surgical procedure performed. 1 A recent systematic review in the obstetrics literature summarized the findings of over 70 randomized controlled trials (RCTs), 10 meta-analyses, and 12 Cochrane reviews of each technical aspect of CD. 2 Compared with women who deliver vaginally, those who deliver by CD have increased morbidity and mortality. 3

Not only is CD one of the most common surgeries performed worldwide, it is also one of the most unique as there are two patients to consider—the mother and the neonate. Whether performed in an urgent or nonurgent setting, atraumatic delivery of a live, vigorous neonate and subsequent minimization of maternal morbidity remains paramount and inherent in all surgical considerations. The objective of this review is to provide a summary of surgical techniques of the procedure and review the evidence basis for them for the nonobstetrician.

## Indications for Cesarean Delivery

The reasons to deliver via CD are multiple and varied depending on the circumstances of a woman's obstetric history and current pregnancy. Women with a history of previous CD, after appropriate counseling, may choose to undergo another CD in subsequent pregnancies and repeat CD accounts for a large proportion of CD indications. For example, the Consortium on Safe Labor in the United States found that a previous uterine scar was the primary indication for over half of all CDs and that 83% of women with a uterine scar are delivered by CD. 4 In contrast, Table 1 summarizes the most common indications for CD in women undergoing primary CD. 5 6

**Table 1:** Most common indications for primary cesarean delivery 5 6

Indication for primary cesarean	Percent
Labor arrest	34
Nonreassuring fetal tracing	23
Malpresentation	17
Multiple gestation	7
Maternal-fetal	5
Macrosomia	4
Preeclampsia	3
Maternal request	3
Other obstetric indications	4

## Evidence-Based Technical Aspects of Cesarean Delivery

Over 220 RCTs published since 1960 have been performed with regard to specific surgical techniques of CD or various generalized surgical approaches to operative technique. 2 7 The generalized surgical approaches that have been compared in RCTs include the Pfannenstiel-Kerr method, Joel-Cohen method, Misgav-Ladach method, and Modified Misgav-Ladach method. 8 9 10 11 Table 2 summarizes the specific techniques of these generalized approaches. In general, blunt entry reduces operative time compared with sharp entry. In addition, short-term outcomes such as blood loss, fever, and postoperative pain are reduced in those techniques using Joel-Cohen techniques. None of the RCTs provided sufficient data to assess neonatal morbidity or the long-term maternal morbidity.

**Table 2:** Summary of generalized CD surgical approaches

	PKM	JCM	MLM	MMLM
Abdominal entry				
Skin	Pfannenstiel	Joel-Cohen	Joel-Cohen	Pfannenstiel
Subcutaneous	Sharp dissection	Blunt dissection	Blunt dissection	Blunt dissection
Fascia	Sharp extension	Blunt extension	Blunt extension	Blunt extension
Peritoneum	Sharp entry	Blunt entry	Blunt entry	Blunt entry
Uterine entry				
Hysterotomy	Sharp superficial, then blunt entry	Sharp superficial, then blunt entry	Sharp superficial, then blunt entry	Sharp superficial, then blunt entry
Placenta removal	Manual	Spontaneous	Manual	Spontaneous
Hysterotomy closure	Single layer, interrupted	Single layer, interrupted	Single layer, running	Single layer, running
Abdominal closure				
Peritoneum	Closed	Not closed	Not closed	Closed
Fascia	Interrupted	Interrupted	Continuous	Continuous
Subcutaneous	Not sutured	Not sutured	Not sutured	Not sutured
Skin	Continuous	Continuous	Mattress	Continuous

Abbreviations: CD, cesarean delivery; JCM, Joel-Cohen method; MLM, Misgav-Ladach method; MMLM, Modified Misgav-Ladach method; PKM, Pfannenstiel-Kerr method.

Note: Some studies report slight variations to these techniques.

In addition to the aforementioned generalized CD techniques, two of the largest RCTs that evaluated optimal surgical techniques combined several steps and are worth mentioning. The CAESAR study collaborative randomized over 3,000 women to three specific technical steps: (1) single- versus double-layer uterine incision closure, (2) peritoneum closure versus nonclosure, and (3) liberal versus restricted subrectus sheath drainage. 12 There were no statistical differences of the primary outcome of maternal morbidity from infection or secondary short-term outcomes among any of the techniques utilized. The CORONIS Collaborative was a multicenter, international RCT of ∼16,000 women who were randomized to include three of five of the following techniques: (1) blunt versus sharp abdominal entry, (2) uterine exteriorization versus in situ hysterotomy repair, (3) single- versus double-layer uterine incision closure, (4) peritoneum closure versus nonclosure, (5) chromic catgut versus polyglactin-910 for uterine repair. 13 Similarly, the short-term adverse outcomes such as death, maternal infectious morbidity, further operative procedures, or blood transfusion (>1 U) did not differ among any of the techniques compared. Notably, the long-term outcomes of most clinical interest such as scar tissue formation (peritoneal closure) and uterine rupture risk (single- or double-layer uterine closure) have not been reported to date.

### Preoperative Considerations

Prior to CD, the following preparation has been evaluated in RCTs: prophylactic antibiotics (7 RCTs), thromboprophylaxis (3 RCTs), preoperative vaginal preparation (2 RCTs), skin preparation (Cochrane review), and indwelling bladder catheterization (2 RCTs). There is insufficient evidence to recommend the optimal type of preoperative skin preparation. Prophylaxis with a single dose of ampicillin or first-generation cephalosporin administered prior to skin incision, however, provides the greatest reduction of maternal morbidity (e.g., endometritis, total morbidity from infection) with no difference in neonatal morbidity (e.g., neonatal sepsis or neonatal intensive care unit admission). 14

In contrast, the RCTs that have evaluated thromboprophylaxis are largely underpowered to make specific recommendations. General hospital policies may dictate routine intermittent compression (mechanical) stockings for all CDs, and universal pharmacologic prophylaxis to reduce the risk of CD-associated venous thromboembolism (VTE) remains vastly understudied. Approximately 10% of maternal deaths in the United States are associated with VTE, and the estimated CD-associated VTE rate is approximately 0.23%, twice the rate as vaginal delivery, highlighting the priority of a well-designed, appropriately powered trial in this population. 15 16

Similar to mechanical stocking use, indwelling bladder catheterization remains a virtually universal practice prior to CD. However, recent data suggests that compared with noncatheterization or immediate removal, there may be a higher incidence of urinary tract infections with no significant difference in urinary retention complications in those who have indwelling bladder catheters placed. 17 As such, there is currently no strong evidence to recommend any of these practices over another.

Although less routinely performed, preoperative vaginal preparation with povidone-iodine scrub is a technique that has demonstrated a reduction in postcesarean endometritis, particularly in women with ruptured membranes. 18 If future studies confirm these findings, the strength of recommendation for this practice may become stronger.

### Intraoperative Considerations

The types of skin incisions available to the surgeon performing CD include midline vertical or low transverse incisions. For purposes of improved cosmesis, decreased postoperative pain, and faster overall recovery, low transverse incisions are generally preferred. Although skin incision type has not individually been compared in an RCT, the Pfannenstiel or Joel-Cohen techniques have been compared in trials of generalized CD approaches. 8 9 10 11 The Joel-Cohen incision is straight, 3 cm below the line that joins the anterior superior iliac spines. In contrast, the Pfannenstiel skin incision is slightly more caudad and curved, 2 to 3 cm or two fingers above the symphysis pubis, with the midportion of the incision within the shaved area of the pubic hair. In these studies, the Joel-Cohen-based surgical methods appear to have less blood loss, fewer fevers, and less postoperative pain. It is unclear, however, the extent for which the specific skin incision type contributes to these short-term outcome measures. Subcutaneous tissue, fascial expansion, and peritoneal entry techniques (e.g., blunt versus sharp) into the gravid abdomen have also not been compared in RCTs. As such, recommendations on the optimal entry technique remain unclear and at the discretion of the primary surgeon.

Once the peritoneum is entered, the gravid uterus generally encompasses the entire intra-abdominal visual field. Retraction with a bladder blade and Richardson retractor may aid with visualization of the lower uterine segment. The practice of creating a bladder flap, or dissecting the visceral peritoneum of the bladder off of the lower uterine segment, has been evaluated in three RCTs with findings summarized in a recent meta-analysis. 19 Based on pooled outcome measures, the omission of this technique reduced the skin-to-delivery interval with no differences found for bladder injury, total operating time, blood loss, or hospitalization duration, thus questioning the benefit of routine use of this technique.

**Fig. 1:**
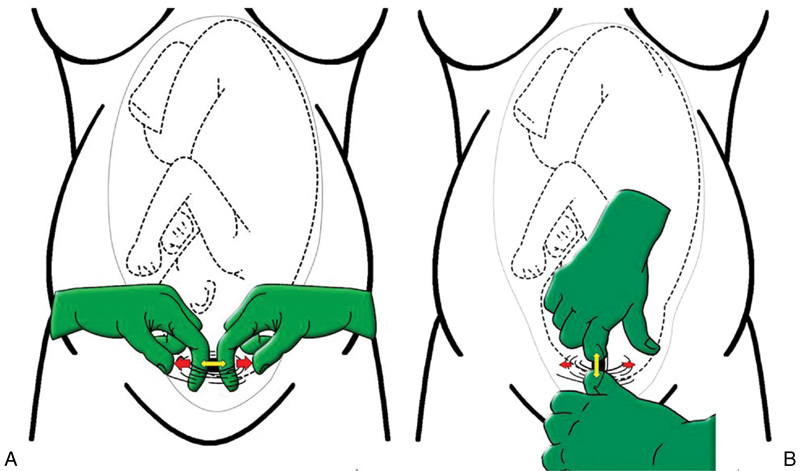
Methods of expansion of the uterine incision. (A) Women in the transversal expansion group had the uterine incision extended by the insertion of both index fingers of the operator into the opening, who then pulled the finger apart laterally and slightly cephalad. (B) In the cephalad–caudad expansion group, a transverse opening of the lower uterine segment was created by separation of the fingers of the surgeon in a cephalad–caudad direction along the midline. (Reused with permission from Cromi A, Ghezzi F, Di Naro E, Siesto G, Loverro G, Bolis P. Blunt expansion of the low transverse uterine incision at cesarean delivery: a randomized comparison of 2 techniques. Am J Obstet Gynecol 2008;199(3):292.e1–292.e6 ^27^ ).

The optimal method of uterine incision and expansion has been evaluated in two RCTs and summarized in a Cochrane review. A 1- to 2-cm incision in the midlower uterine segment may used to enter the uterus. After entry, blunt, cephalad–caudad expansion has been demonstrated to decrease unintended incisional extensions and overall blood loss ( Fig. 1 ). 20

No RCTs have compared delivery techniques of the fetus. The general principles that may assist the delivery provider include: (1) ensuring an adequately sized hysterotomy incision, and (2) when the vertex is engaged in the pelvis, full flexion of the neck (chin to chest) and elevation into the hysterotomy incision. When the vertex is not engaged in the pelvis, the delivery may be accomplished with generous fundal pressure from the assistant, and in some cases delivery requires assistance with either a vacuum or forceps.

Finally, in the case of breech presentation, the following technique is recommended to accomplish atraumatic delivery: (1) grasp one or both feet or, if frank breech, elevate the fetal sacrum out of the hysterotomy; (2) once elevated, rotate the fetal body to sacrum anterior; (3) apply gentle traction parallel to the maternal abdomen to the level of the fetal scapula; (4) sweep both upper extremities with abduction toward the midline; (5) use the Mauriceau-Smellie-Veit maneuver (gentle pressure on the fetal maxilla with the index and middle finger to facilitate flexion of the fetal vertex) to accomplish delivery. There are no RCTs that compare the type of uterine incision (low transverse versus low vertical versus classical) that optimally affects delivery. In general, a low transverse uterine incision should be considered as there is no evidence to suggest difficulty of breech extraction with this type of incision and minimizes the implications a vertical uterine incision has on future pregnancies.

Spontaneous (with gentle cord traction) placental removal compared with manual removal has been evaluated in 6 RCTs. Spontaneous removal is associated with a significant reduction in postoperative endometritis and may also reduce overall blood loss. 21 Once the placenta is removed, the uterus often involutes to a size that may facilitate exteriorization outside of the maternal abdomen. This maneuver can facilitate visualization and repair of the hysterotomy, and based on a meta-analysis of seven RCTs, has similar short-term outcomes of in situ hysterotomy repair. As such, provider preference for this technique is recommended. 22

The optimal postpartum hemorrhage prophylaxis remains an area of active research. Previous RCTs compared oxytocin infusion, oxytocin bolus, misoprostol, carbetocin, and tranexamic acid. These medications, either in combination or individually, have been the subject of 13 RCTs since 2013 and continue to be an active area of study. Currently, oxytocin infusion (10 to 40 U in 1 L of crystalloid infused over 4 to 8 hours) appears to be the optimal medication to prevent postpartum hemorrhage. 2

Hysterotomy closure using single- or double-layer suture closure remains an area of uncertainty. Although retrospective case–control studies have suggested a reduction of uterine rupture in future pregnancies with double-layer suture closure, 2 definitive recommendations cannot be made due to the paucity of RCT data comparing these two options.

Once the hysterotomy is determined to be hemostatic, the surgeon must then turn his or her attention to abdominal closure. If the uterus is exteriorized, it should be returned to its anatomic position. Reassessment of hemostasis once the uterus is replaced is prudent. Intra-abdominal irrigation with warm normal saline to remove blood clots and debris does not appear to reduce morbidity from infection but may increase maternal intraoperative nausea, as evaluated in one RCT. 23

Peritoneal closure compared with nonclosure remains an active topic of research and has resulted in 19 RCTs, 2 meta-analyses, and a systematic review. Evidence supporting closure versus nonclosure depends on the outcome measure studied. However, there is no long-term outcome data on the most clinically relevant outcome measure—adhesion formation. As such, closure versus nonclosure of the peritoneum remains the preference of the surgeon at this time.

Fascial closure is accomplished using absorbable suture in a running fashion. The optimal suture material has not been compared in an RCT. However, blunt-tip needle compared with sharp needle for closure of all tissue layers during CD was evaluated in one RCT and also was included in an analysis in a Cochrane review that also included other types of surgery. Notably, blunt-tip needle use significantly reduces the overall risk of glove perforations and percutaneous exposure incidents and should be routinely available and used in all CDs. 24 25

Subcutaneous skin closure (running or interrupted technique) is recommended if the tissue thickness exceeds 2 cm based on the available evidence of 11 RCTs that compared subcutaneous skin closure versus nonclosure, with or without drain placement. Finally, based on a meta-analysis of 3,112 women in 12 RCTs, skin closure with absorbable suture rather than metal staples is strongly recommended as this method significantly decreases wound morbidity (particularly wound separation) without a difference noted in pain, patient satisfaction, or cosmetic results. 26

## Summary

**Fig. 2:**
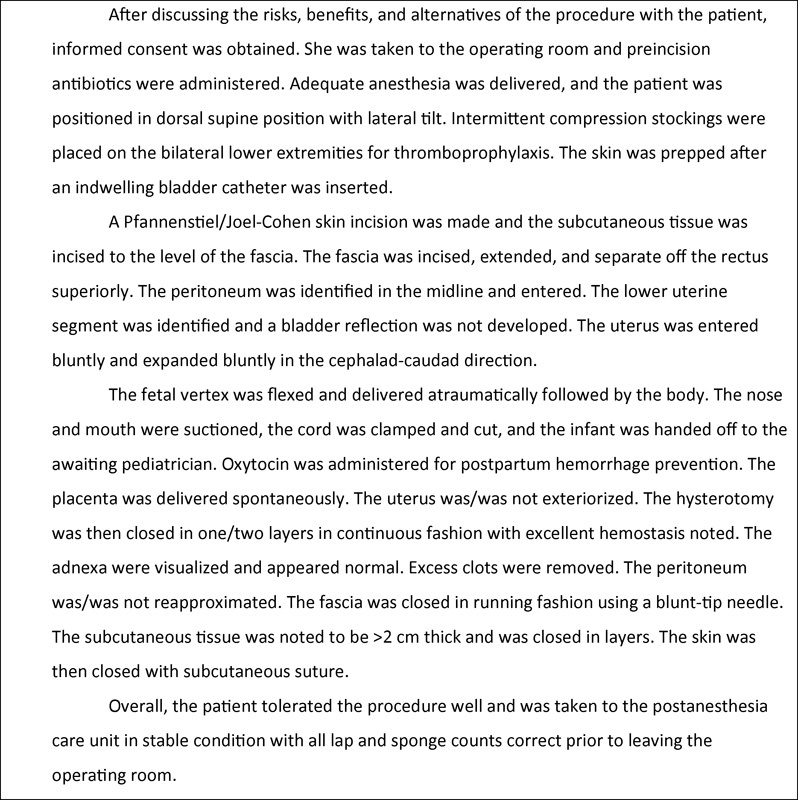
Sample cesarean delivery operative report inclusive of evidence-based techniques.

CD remains the most common major abdominal surgery performed in the United States. As such, the surgical techniques evaluated in well-designed RCTs that demonstrate maternal benefit should be incorporated into practice. Table 3 summarizes the techniques with the strongest evidence basis for 11 steps of CD that should be commonly performed when feasible, and Fig. 2 provides an example of a sample operative report that incorporates the evidence-based surgical approaches reviewed herein. Although over 170 RCTs have been performed regarding optimizing this common procedure and the results are encouraging, further work is needed to minimize surgical morbidity. Specifically, the optimal methods for postpartum hemorrhage prevention, adhesion prevention, and VTE prophylaxis have not been determined and represent areas that could markedly improve maternal morbidity and mortality. If evidence of a surgical technique appears preferred over another, obstetricians and nonobstetricians alike should be comfortable adopting the evidence-based techniques when performing and teaching CD.

**Table 3:** Evidence-based cesarean delivery techniques with strong recommendations

Technique	Recommendation
Pre- and intraoperative preparation	
Prophylactic antibiotics Postpartum hemorrhage prevention	Single dose, ampicillin or first-generation cephalosporin prior to skin incision Oxytocin infusion (10–40 IU in 1 L crystalloid over 4–8 h)
Abdominal entry	
Skin incision Subcutaneous and fascial incision	Low transverse incision (Pfannenstiel or Joel-Cohen a ) Surgeon preference for blunt or sharp expansion
Uterine considerations	
Expansion of uterine incision Placental removal Uterine exteriorization Uterine closure	Blunt, cephalad–caudad direction Spontaneous Surgeon preference One layer if future fertility undesired
Abdominal closure	
Needle type Subcutaneous closure Skin closure	Blunt tip needles Suture closure if ≥2 cm in depth Suture closure

a Joel-Cohen incision is straight, 3 cm below the line that joins the anterior superior iliac spines, slightly more cephalad than Pfannenstiel. Pfannenstiel skin incision is slightly curved, 2–3 cm or two fingers above the symphysis pubis, with the midportion of the incision within the shaved area of the pubic hair.
